# Supplementary material for: Genome‐wide association analysis of hyperspectral reflectance data to dissect the genetic architecture of growth‐related traits in maize under plant growth‐promoting bacteria inoculation
Source: Plant Direct. 2023 Apr 24;7(4):e492. doi: 10.1002/pld3.492 (PMC10123960; doi:10.1002/pld3.492)
Supplement: Supplementary file 1 — PLD3_492_SupplementalMaizeHyperspectralGWAS.pdf [file PLD3-7-e492-s001.pdf]

Genome-wide association analysis of hyperspectral reflectance data to dissect the genetic architecture of growth-related traits in maize under plant growth-promoting bacteria inoculation

Rafael Massahiro Yassue[1], Giovanni Galli[1], Chunpeng James Chen[2,3], Roberto Fritsche-Neto[1,4,*], and Gota Morota[2,3,*]

[1]Department of Genetics, 'Luiz de Queiroz' College of Agriculture, University of São Paulo, São Paulo, Brazil

[2]School of Animal Sciences, Virginia Polytechnic Institute and State University, Blacksburg, USA

[3]Center for Advanced Innovation in Agriculture, Virginia Polytechnic Institute and State University, Blacksburg, VA 24061 USA

[4]Quantitative Genetics and Biometrics Cluster, International Rice Research Institute, Los Baños, Philippines

Supplementary Tables

Table S1: List of hyperspectral indices used in the study.

Index	Abbreviation	Formula	Reference
Boochs index	Boochs	D_{703}	(Boochs et al., 1990)
Boochs index 2	Boochs2	D_{720}	(Boochs et al., 1990)
Carter index	Carter	R_{695}/R_{420}	(Carter, 1994)
Carter index 2	Carter2	R_{695}/R_{760}	(Carter, 1994)
Carter index 3	Carter3	R_{605}/R_{760}	(Carter, 1994)
Carter index 4	Carter4	R_{710}/R_{760}	(Carter, 1994)
Carter index 5	Carter5	R_{695}/R_{670}	(Carter, 1994)
Carter index 5	Carter6	R_{550}	(Carter, 1994)
Chlorophyll index	CI	$R_{675} * R_{690}/R_{683}^2$	(Zarco-Tejada et al., 2013)
Chlorophyll index 2	CI2	$R_{760}/R_{700} - 1$	(Gitelson et al., 2003)
Chlorophyll absorption integral	ClAInt	$\int_{60n\ \mathrm{nm}}^{735\ \mathrm{nm}} \mathrm{R}$	(Oppelt and Mauser, 2004)
Carotenoid reflectance index 1	CRI1	$1/R_{515} - 1/R_{550}$	(Gitelson et al., 2003)
Carotenoid reflectance Iindex 2	CRI2	$1/R_{515} - 1/R_{770}$	(Gitelson et al., 2003)
Carotenoid reflectance index 3	CRI3	$1/R_{515} - 1/R_{550} * R_{770}$	(Gitelson et al., 2003)
Carotenoid reflectance index 4	CRI4	$1/R_{515} - 1/R_{700} * R_{770}$	(Gitelson et al., 2003)
Simple Ratio 730/706	D1	D_{730}/D_{706}	(Zarco-Tejada et al., 2013)
Simple Ratio 705/722	D2	D_{705}/D_{722}	(Zarco-Tejada et al., 2013)
Datt index	Datt	$(R_{850} - R_{710})/(R_{850} - R_{680})$	(Datt, 1999)
Datt index2	Datt2	R_{850}/R_{710}	(Datt, 1999)
Datt index 3	Datt3	D_{754}/D_{704}	(Datt, 1999)
Datt index 4	Datt4	$R_{672}/(R_{550} * R_{708})$	(Datt, 1999)
Datt index 5	Datt5	R_{672}/R_{550}	(Datt, 1999)
Datt index 6	Datt6	$(R_{860})/(R_{550} * R_{708})$	(Datt, 1999)
Double difference index	DD	$(R_{749} - R_{720}) - (R_{701} - R_{672})$	(le Maire et al., 2004)
New double difference index	DDn	$2 * (R_{710} - R_{660} - R_{760})$	(le Maire et al., 2008)
Double peak index	DPI	$(D_{688} * D_{710})/D_{697}^2$	(Zarco-Tejada et al., 2013)
Disease water stress index	DWSI4	R_{550}/R_{680}	(Apan et al., 2004)
Normalized ratio between the maxima of the first derivatives of reflectances at the red edge and green regions	EGFN	$(\max(D_{650:750}) - \max(D_{500:550}))/(\max(D_{650:750}) + \max(D_{500:550}))$	(Peñuelas et al., 1994)
Ratio between dRE and dG	EGFR	$\max(D_{650:750})/\max(D_{500:550})$	(Peñuelas et al., 1994)
Enhanced vegetation index	EVI	$25 * ((R_{800} - R_{670})/(R_{800} - (6 * R_{670}) - (75 * R_{475}) + 1))$	(Huete et al., 1997)
Generalized difference vegetation index	GDVI_2	$(R_{800}^n - R_{680}^n)/(R_{800}^n + R_{680}^n)$	(Wu, 2014)
Generalized difference vegetation index	GDVI_3		(Wu, 2014)
Generalized difference vegetation index	GDVI_4		(Wu, 2014)
Greenness index	GI	R_{554}/R_{677}	(Smith et al., 1995)
Gitelson index	Gitelson	$1/R_{700}$	(Gitelson et al., 1999)
Gitelson index 2	Gitelson2	$(R_{750} - R_{800}/R_{695} - R_{740}) - 1$	(Gitelson et al., 2003)

Rxxx: Reflectance at wavelength 'xxx'.
Dxxx: First derivation of reflectance values at wavelength 'xxx'.
maxxxx: maximum value at wavelength 'xxx'.

Table S1: (Continued). List of hyperspectral indices used in the study.

Index	Abbreviation	Formula	Reference
Maccioni index	Maccioni	$(R_{780} - R_{710})/(R_{780} - R_{680})$	(Maccioni et al., 2001)
Modified chlorophyll absorption in reflectance index	MCARI	$((R_{700} - R_{670}) - 0.2 * (R_{700} - R_{550})) * (R_{700}/R_{670})$	(Daughtry et al., 2000)
	MCARI/OSAVI		(Daughtry et al., 2000)
Modified chlorophyll absorption in reflectance index 2	MCARI2	$((R_{750} - R_{705}) - 0.2 * (R_{750} - R_{550})) * (R_{750}/R_{705})$	(Wu et al., 2008)
	MCARI2/OSAVI2		(Wu et al., 2008)
Modified normalized difference at 705 nm wavelength	mND705	$(R_{750} - R_{705})/(R_{750} + R_{705} - 2 * R_{445})$	(Sims and Gamon, 2002)
Modified normalized difference vegetation index	mNDVI	$(R_{800} - R_{680})/(R_{800} + R_{680} - 2 * R_{445})$	(Sims and Gamon, 2002)
Normalized difference physiological reflectance index	MPRI	$(R_{515} - R_{530})/(R_{515} + R_{530})$	(Hernández-Clemente et al., 2011)
Modified red-edge inflection point	mREIP	red-edge inflection point using Gaussain fit	(MILLER et al., 1990)
Modified soil-adjusted vegetation index	MSAVI	$05 * (2 * R_{800} + 1 - ((2 * R_{800} + 1)^2 - 8 * (R_{800} - R_{670}))^{05})$	(Qi et al., 1994)
Modified simple ratio of reflectance	mSR	$(R_{800} - R_{445})/(R_{680} - R_{445})$	(Sims and Gamon, 2002)
Modified simple ratio of reflectance 2	mSR2	$(R_{750}/R_{705}) - 1/(R_{750}/R_{705} + 1)^{05}$	(Chen, 1996)
Modified simple ratio of reflectance 705	mSR705	$(R_{750} - R_{445})/(R_{705} - R_{445})$	(Sims and Gamon, 2002)
MERIS terrestrial chlorophyll index	MTCI	$(R_{754} - R_{709})/(R_{709} - R_{681})$	(Dash and Curran, 2004)
Modified triangular vegetation index	MTVI	$1.2 * (1.2 * (R_{800} - R_{550}) - 25 * (R_{670} - R_{550}))$	(Haboudane et al., 2002)
Chlorophyll absorption ratio index	Cari		(Kim et al., 1994)
Normalized difference vegetation index	NDVI	$(R_{800} - R_{680})/(R_{800} + R_{680})$	(Tucker, 1979)
Normalized difference vegetation index 2	NDVI2	$(R_{750} - R_{705})/(R_{750} + R_{705})$	(Gitelson and Merzlyak, 1994)
Normalized difference vegetation index 3	NDVI3	$(R_{682} - R_{553})/(R_{682} + R_{553})$	(Gandia et al., 2004)
Normalized pigment chlorophyll index	NPCI	$(R_{680} - R_{430})/(R_{680} + R_{430})$	(Peñuelas et al., 1994)
Optimized soil-adjusted vegetation index	OSAVI	$(1+0.16)*(R_{800}-R_{670})/(R_{800}+R_{670} + 0.16)$	(Rondeaux et al., 1996)
Optimized soil-adjusted vegetation index 2	OSAVI2	$(1+0.16)*(R_{750}-R_{705})/(R_{750}+R_{705} + 0.16)$	(Wu et al., 2008)
Ratio analysis of reflectance spectra	PARS	R_{746}/R_{513}	(Chappelle et al., 1992)
Photochemical reflectance index	PRI	$(R_{531} - R_{570})/(R_{531} + R_{570})$	(Gamon et al., 1992)
Photochemical reflectance index normalized	PRI_norm	$PRI*(-1)/(RDVI*R_{700}/R_{670})$	(Zarco-Tejada et al., 2013)
	PRI * CI2	$PRI * CI2$	(Garrity et al., 2011)
Plant senescence reflectance index	PSRI	$(R_{678} - R_{500}/R_{750}$	(Merzlyak et al., 1999)

Rxxx: Reflectance at wavelength 'xxx'.
Dxxx: First derivation of reflectance values at wavelength 'xxx'.
maxxxx: maximum value at wavelength 'xxx'.

Table S1: (Continued). List of hyperspectral indices used in the study.

Index	Abbreviation	Formula	Reference
Red-edge position linear interpolation	REP_LE	Red-edge position through linear extrapolation	(Cho and Skidmore, 2006)
Red-edge position linear interpolation	REP_Li	$R_{re} = (R_{670} + R_{780})/2$	(Guyot and Baret, 1988)
Soil adjusted vegetation index	SAVI	$(1 + L) * (R_{800} - R_{670})/(R_{800} + R_{670} + L)$	(Huete, 1988)
Structure insensitive pigment index	SIPI	$(R_{800} - R_{445})/(R_{800} - R_{680})$	(Peñuelas et al., 1995; PEN¯UELAS et al., 1995)
spectral polygon vegetation index	SPVI	$0.4 * 3.7 * (R_{800} - R_{670}) - 1.2 * ((R_{530} - R_{670})^2)^{0.5}$	(Vincini et al., 2006)
Simple ratio	SR	R_{800}/R_{680}	(Jordan, 1969)
Simple ratio 1	SR1	R_{750}/R_{700}	(Gitelson and Merzlyak, 1997)
Simple ratio 2	SR2	R_{752}/R_{690}	(Gitelson and Merzlyak, 1997)
Simple ratio 3	SR3	R_{750}/R_{550}	(Gitelson and Merzlyak, 1997)
Simple ratio 4	SR4	R_{700}/R_{670}	(McMurtrey et al., 1994)
Simple ratio 5	SR5	R_{675}/R_{700}	(Chappelle et al., 1992)
Simple ratio 6	SR6	R_{750}/R_{710}	(Zarco-Tejada and Miller, 1999)
Simple ratio 7	SR7	R_{440}/R_{690}	(Lichtenthaler et al., 1996)
Simple ratio 8	SR8	R_{515}/R_{550}	(Hernández-Clemente et al., 2012)
Simple ratio pigment index	SRPI	R_{430}/R_{680}	(PEN¯UELAS et al., 1995)
Sum of first derivative reflectance 1	Sum_Dr1	$\Sigma_{i=626}^{795} D1_i$	(Elvidge and Chen, 1995)
Sum of first derivative reflectance 2	Sum_Dr2	$\Sigma_{i=680}^{780} D1_i$	(Filella and Peñuelas, 1994)
Transformed chlorophyll absorption reflectance index	TCARI	$3 * ((R_{700} - R_{670}) - 02 * R_{700} - R_{550}) * (R_{700}/R_{670}))$	(Haboudane et al., 2002)
	TCARI/OSAVI	$TCARI/OSAVI$	(Haboudane et al., 2002)
Transformed chlorophyll absorption reflectance index 2	TCARI2	$3 * ((R_{750} - R_{705}) - 02 * (R_{750} - R_{550}) * (R_{750}/R_{705}))$	(Wu et al., 2008)
Triangular greenness index	TGI	$-0.5(190(R_{670} - R_{550}) - 1.20(R_{670} - R_{480}))$	(Hunt et al., 2013)
Triangular vegetation index	TVI	$0.5 * (120 * (R_{750} - R_{550}) - 200 * (R_{670} - R_{550}))$	(Broge and Leblanc, 2001)
Vogelmann index	Vogelmann	R_{740}/R_{720}	Vogelmann et al. (1993)
Vogelmann index 2	Vogelmann2	$(R_{734} - R_{747})/(R_{715} + R_{726})$	Vogelmann et al. (1993)
Vogelmann index 3	Vogelmann3	D_{715}/D_{705}	Vogelmann et al. (1993)
Vogelmann index 4	Vogelmann4	$(R_{734} - R_{747})/(R_{715} + R_{720})$	Vogelmann et al. (1993)
Ratio vegetation index	RVI	$R790/R650$	(Pearson and Miller, 1972)

Rxxx: Reflectance at wavelength 'xxx'.
Dxxx: First derivation of reflectance values at wavelength 'xxx'.
maxxxx: maximum value at wavelength 'xxx'.

Table S1: (Continued). List of hyperspectral indices used in the study.

Index	Abbreviation	Formula	Reference
Red-edge position linear interpolation	REP	$R_{700} + 40 * ((R_{670} + R_{780})/2 - R_{700})/(R_{740} - R_{700}))$	(Ali and Imran, 2020)
First derivative	FD730	D_{730}	(Peng et al., 2018)
Modified Datt index	MDATT	$(R_{719} - R_{726})/(R_{719} - R_{743})$	(Velichkova and Krezhova, 2019)
Modified red-edge simple ratio	MRESR	$(R_{750} - R_{450})/(R_{705} + R_{450})$	(Velichkova and Krezhova, 2019)
Near infrared/Red 1	NR1	R_{760}/R_{695}	(Velichkova and Krezhova, 2019)
Near infrared/Red 2	NR2	R_{800}/R_{650}	(Velichkova and Krezhova, 2019)
Zarco - Miller Index	ZM	R_{750}/R_{710}	(Velichkova and Krezhova, 2019)
Greenness	G	R_{554}/R_{667}	(Velichkova and Krezhova, 2019)
Blue/Green	BG	R_{677}/R_{554}	(Velichkova and Krezhova, 2019)
Vogelmann red edge	VREI1	R_{740}/R_{720}	(Velichkova and Krezhova, 2019)
Single band index	SB	$1/R_{700}$	(Velichkova and Krezhova, 2019)
Single-difference index	SD	$(1/R_{550}) - (1/R_{750})$	(Velichkova and Krezhova, 2019)
Green Chl index	Chlg	$R_{760}/R_{550} - 1$	(Velichkova and Krezhova, 2019)
Red edge Chl index	Chlr	$R_{760}/R_{714} - 1$	(Velichkova and Krezhova, 2019)
Simple ratio pigment specific simple ratio (Cholophyll a)	PSSRa	R_{800}/R_{675}	(Blackburn, 1998a)
Simple ratio pigment specific simple ratio B1	PSSRb	R_{800}/R_{650}	(Blackburn, 1998a)
Normalized pheophytinization index	NPQI	$(R_{415} - R_{435})/(R_{415} + R_{435})$	(PEN¯UELAS et al., 1995)
Normalized difference nitrogenindex	NDNI	$(R_{415} - R_{435})/(R_{415} + R_{435})$	(Wang and Wei, 2016)
	MLO	R_{531}/R_{645}	(Meiforth et al., 2020)
Lichtenthaler index	LIC	$(R_{850} - R_{710})/(R_{850} + R_{680})$	(Lichtenthaler et al., 1996)
Lichtenthaler index 1	LIC1	$(R_{800} - R_{680})/(R_{800} + R_{680})$	(Lichtenthaler et al., 1996)
Lichtenthaler index 2	LIC2	R_{440}/R_{690}	(Lichtenthaler et al., 1996)
Blue/Green pigment index	BGI	R_{450}/R_{550}	(ZARCOTEJADA et al., 2005)
Blue/Red pigment index	BRI	(R_{450}/R_{690})	(ZARCOTEJADA et al., 2005)
Ratio analysis of reflectance spectra chlorophyll a	RARSa	(R_{675}/R_{700})	(Kycko et al., 2019)
Ratio analysis of reflectance spectra chlorophyll b	RARSb	$(R_{675}/(R_{650} * R_{700}))$	(Montesinos-López et al., 2017)

Rxxx: Reflectance at wavelength 'xxx'.
Dxxx: First derivation of reflectance values at wavelength 'xxx'.
maxxxx: maximum value at wavelength 'xxx'.

Table S1: (Continued). List of hyperspectral indices used in the study.

Index	Abbreviation	Formula	Reference
Gitelson and Merzylak index 1	GMI1	R_{750}/R_{550}	(Gitelson et al., 2003)
Gitelson and Merzylak index2	GMI2	R_{750}/R_{700}	(Gitelson et al., 2003)
Green normalized difference vegetation indexx	GreenNDVI	$(R_{800} - R_{550})/(R_{800} + R_{550})$	(Gitelson et al., 1996)
Simple ratio 800/635 pigment specific simple ratio (Cholophyll b)	PSSR	R_{800}/R_{635}	(Blackburn, 1998b)
Pigment specific normalised difference	PSND	$(R_{800} - R_{470})/(R_{800} - R_{470})$	(Blackburn, 1998b)
Plant water index	PWI	R_{900}/R_{970}	(Peñuelas et al., 1994)
Renormalized difference vegetation index	RDVI	$(R_{800} - R_{670})/\sqrt{R_{800} + R_{670}}$	(Roujean and Breon, 1995)
Zhen Index	Zen	$(R_{785} - R_{810})/((R_{785} + R_{810}) - (2 * R_{802}))$	(Zhen et al., 2020)
Difference vegetation index	DVI	R_{782}/R_{675}	(Peng et al., 2018)
Transformed soil adjusted vegetation index	TSAVI	$0.5 * (R_{782} - (0.5 * R_{675}) - 0.2)/((0.5 * R_{782}) + (0.5 * R_{675}) - 01)$	(Baret et al., 1989)
Perpendicular vegetation index	PVI	$(R_{800} - (0.2 * R_{670}) - 06)/1.019)$	(Darvishzadeh et al., 2006)
Ratio analysis of reflectance spectra chlorophyll c	RARSc	(R_{760}/R_{500})	(Montesinos-López et al., 2017)
Green normalized difference vegetation index	GNDVI	$(R_{780} - R_{670})/(R_{780} + R_{670})$	(Montesinos-López et al., 2017)
Red normalized difference vegetation index	RNDVI	$(R_{780} - R_{550})/(R_{780} + R_{550})$	(Montesinos-López et al., 2017)

Rxxx: Reflectance at wavelength 'xxx'.
Dxxx: First derivation of reflectance values at wavelength 'xxx'.
maxxxx: maximum value at wavelength 'xxx'.

Table S2: Selected single nucleotide polymorphisms based on BayesC using the posterior inclusion probability threshold of 0.10 for plant height (PH) under the B- management.

Management	Trait	Chromosome	Marker ID	Minor allele frequency	Posterior inclusion probability
B-	PH	1	Chr1_6916155	0.38	0.814
B-	PH	1	Chr1_115455586	0.12	0.264
B-	PH	2	Chr2_14984692	0.44	0.277
B-	PH	2	Chr2_43718939	0.12	0.162
B-	PH	2	Chr2_108275267	0.47	0.112
B-	PH	2	Chr2_112068617	0.49	0.128
B-	PH	2	Chr2_112233172	0.49	0.291
B-	PH	3	Chr3_57358057	0.31	0.733
B-	PH	4	Chr4_162995251	0.29	0.387
B-	PH	4	Chr4_174621437	0.33	0.215
B-	PH	4	Chr4_214178316	0.36	0.133
B-	PH	4	Chr4_238615869	0.26	0.677
B-	PH	5	Chr5_4105099	0.32	0.143
B-	PH	5	Chr5_17293217	0.28	0.137
B-	PH	6	Chr6_93020758	0.08	0.122
B-	PH	6	Chr6_168918815	0.33	0.190
B-	PH	7	Chr7_161417373	0.30	0.187
B-	PH	8	Chr8_67750993	0.12	0.172
B-	PH	9	Chr9_28676437	0.23	0.217
B-	PH	9	Chr9_28678507	0.22	0.211
B-	PH	9	Chr9_32797796	0.49	0.146
B-	PH	9	Chr9_32797855	0.48	0.210
B-	PH	9	Chr9_150486056	0.30	0.176
B-	PH	10	Chr10_133638870	0.40	0.139

Table S3: Selected single nucleotide polymorphisms based on BayesC using the posterior inclusion probability threshold of 0.10 for stalk diameter (SD) under the B- management.

Management	Trait	Chromosome	Marker ID	Minor allele frequency	Posterior inclusion probability
B-	SD	1	Chr1_203994793	0.49	0.109
B-	SD	2	Chr2_10595188	0.45	0.102
B-	SD	2	Chr2_189507598	0.40	0.156
B-	SD	2	Chr2_220144531	0.42	0.144
B-	SD	3	Chr3_133566445	0.21	0.108
B-	SD	3	Chr3_157011824	0.34	0.133
B-	SD	5	Chr5_53086536	0.41	0.109
B-	SD	7	Chr7_5874773	0.13	0.103
B-	SD	9	Chr9_45942495	0.37	0.203

Table S4: Selected single nucleotide polymorphisms based on BayesC using the posterior inclusion probability threshold of 0.10 for plant height (PH) under the B+ management.

Management	Trait	Chromosome	Marker ID	Minor allele frequency	Posterior inclusion probability
B+	PH	1	Chr1_6540758	0.37	0.114
B+	PH	1	Chr1_200165533	0.37	0.210
B+	PH	1	Chr1_255269329	0.36	0.206
B+	PH	2	Chr2_129846748	0.40	0.265
B+	PH	2	Chr2_169473601	0.49	0.397
B+	PH	3	Chr3_158981956	0.22	0.109
B+	PH	4	Chr4_162995251	0.29	0.101
B+	PH	4	Chr4_176216697	0.15	0.114
B+	PH	4	Chr4_203497008	0.19	0.156
B+	PH	5	Chr5_3760644	0.45	0.412
B+	PH	5	Chr5_3760660	0.39	0.115
B+	PH	5	Chr5_75194167	0.18	0.137
B+	PH	5	Chr5_195847141	0.48	0.283
B+	PH	6	Chr6_108027757	0.49	0.121
B+	PH	6	Chr6_150545871	0.47	0.387
B+	PH	6	Chr6_168918815	0.33	0.239
B+	PH	7	Chr7_99946782	0.15	0.209
B+	PH	8	Chr8_155019631	0.23	0.228
B+	PH	10	Chr10_144153912	0.25	0.230
B+	PH	10	Chr10_145449487	0.20	0.144
B+	PH	10	Chr10_145449559	0.21	0.128

Table S5: Selected single nucleotide polymorphisms based on BayesC using the posterior inclusion probability of 0.10 for stalk diameter (SD) under the B+ management.

Management	Trait	Chromosome	Marker ID	Minor allele frequency	Posterior inclusion probability
B+	SD	1	Chr1_17324463	0.31	0.118
B+	SD	1	Chr1_49402080	0.27	0.120
B+	SD	1	Chr1_203994793	0.49	0.196
B+	SD	1	Chr1_286728045	0.47	0.149
B+	SD	2	Chr2_16421052	0.34	0.166
B+	SD	2	Chr2_32734863	0.26	0.143
B+	SD	2	Chr2_32734876	0.25	0.190
B+	SD	2	Chr2_38816460	0.26	0.306
B+	SD	2	Chr2_82458517	0.45	0.303
B+	SD	2	Chr2_169026437	0.36	0.447
B+	SD	2	Chr2_219293526	0.36	0.241
B+	SD	3	Chr3_205139465	0.29	0.113
B+	SD	4	Chr4_72636153	0.30	0.657
B+	SD	4	Chr4_164089694	0.10	0.119
B+	SD	4	Chr4_169305835	0.19	0.115
B+	SD	4	Chr4_193981325	0.29	0.112
B+	SD	4	Chr4_244379137	0.45	0.120
B+	SD	6	Chr6_2916367	0.16	0.114
B+	SD	6	Chr6_99963274	0.23	0.100
B+	SD	6	Chr6_103106825	0.12	0.118
B+	SD	7	Chr7_41779617	0.26	0.193
B+	SD	7	Chr7_41779668	0.37	0.563
B+	SD	7	Chr7_161417373	0.30	0.140
B+	SD	8	Chr8_151688268	0.29	0.102
B+	SD	8	Chr8_167863049	0.44	0.122
B+	SD	9	Chr9_18547071	0.20	0.115
B+	SD	10	Chr10_95462636	0.33	0.162

Table S6: Selected single nucleotide polymorphisms based on BayesC using the posterior inclusion probability threshold of 0.10 for shoot dry mass (SDM) under the B+ management.

Management	Trait	Chromosome	Marker ID	Minor allele frequency	Posterior inclusion probability
B+	SDM	1	Chr1_26874225	0.27	0.120
B+	SDM	1	Chr1_26874654	0.41	0.103
B+	SDM	2	Chr2_34931185	0.47	0.102
B+	SDM	4	Chr4_181569268	0.33	0.176
B+	SDM	10	Chr10_129809018	0.37	0.110

Table S7: Common single nucleotide polymorphism identified in the current study and Yassue et al. (2021). PH: plant height, SD: stalk diameter, SDM: shoot dry mass

Management	Trait	Chromosome	Marker ID	Minor allele frequency	Posterior inclusion probability
B+	SDM	4	Chr4_181569268	0.33	0.176
B+	SD	2	Chr2_38816460	0.26	0.306
B+	SD	2	Chr2_169026437	0.36	0.447
B+	SD	4	Chr4_72636153	0.30	0.657
B+	SD	6	Chr6_2916367	0.16	0.114
B+	SD	7	Chr7_41779668	0.37	0.563
B+	PH	7	Chr7_99946782	0.15	0.209
B-	SD	9	Chr9_45942495	0.37	0.203
B-	PH	1	Chr1_115455586	0.12	0.264
B-	PH	3	Chr3_57358057	0.31	0.733
B-	PH	5	Chr5_4105099	0.32	0.143

Supplementary Figure

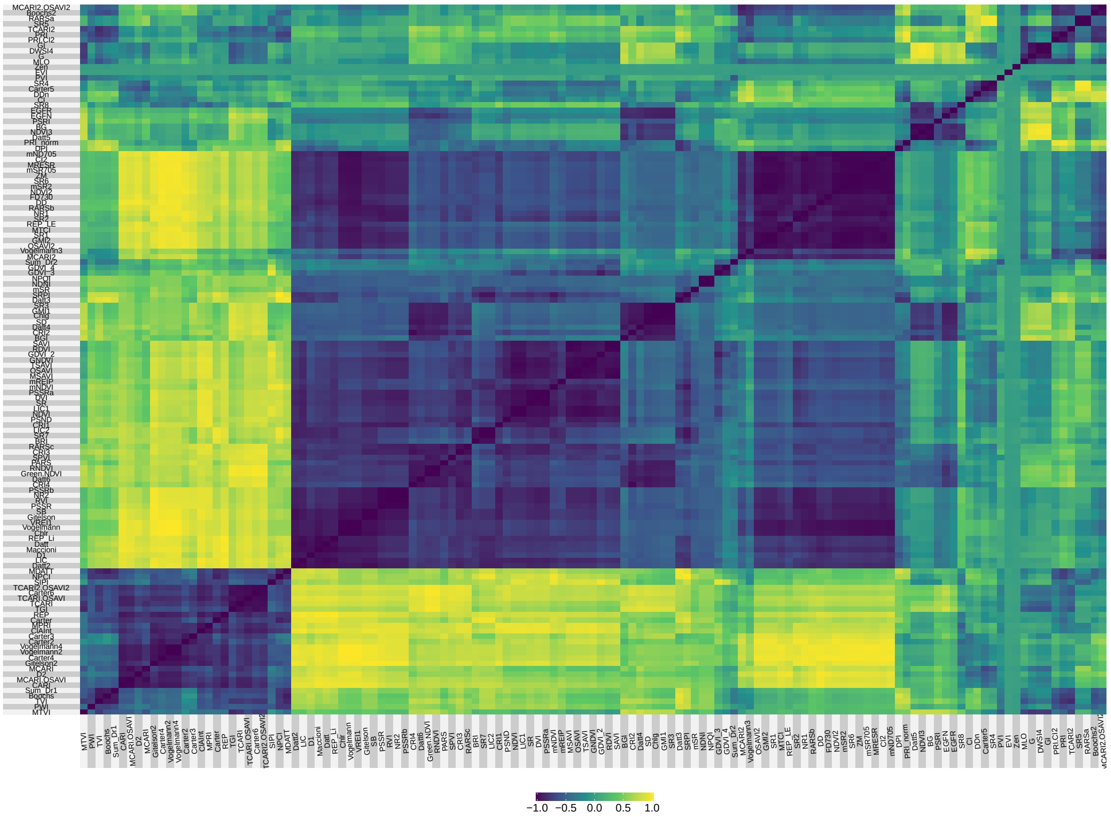

Figure S1: Correlation matrix of 131 hyperspectral indices. The correlation coefficients were computed from the averages of the two managements (B- and B+).

References

Ali, A. and Imran, M. (2020). Evaluating the potential of red edge position (REP) of hyperspectral remote sensing data for real time estimation of LAI & chlorophyll content of kinnow mandarin (citrus reticulata) fruit orchards. *Scientia Horticulturae*, 267:109326.

Apan, A., Held, A., Phinn, S., and Markley, J. (2004). Detecting sugarcane "orange rust" disease using EO-1 Hyperion hyperspectral imagery. *International Journal of Remote Sensing*, 25(2):489–498.

Baret, F., Guyot, G., and Major, D. (1989). TSAVI: A vegetation index which minimizes soil brightness effects on LAI and APAR estimation. In *12th Canadian Symposium on Remote Sensing Geoscience and Remote Sensing Symposium,*. IEEE.

Blackburn, G. A. (1998a). Quantifying chlorophylls and caroteniods at leaf and canopy scales. *Remote Sensing of Environment*, 66(3):273–285.

Blackburn, G. A. (1998b). Quantifying chlorophylls and caroteniods at leaf and canopy scales: An evaluation of some hyperspectral approaches. *Remote Sensing of Environment*, 66(3):273 – 285.

Boochs, F., Kupfer, G., Dockter, K., and Kühbauch, W. (1990). Shape of the red edge as vitality indicator for plants. *International Journal of Remote Sensing*, 11(10):1741–1753.

Broge, N. and Leblanc, E. (2001). Comparing prediction power and stability of broadband and hyperspectral vegetation indices for estimation of green leaf area index and canopy chlorophyll density. *Remote Sensing of Environment*, 76(2):156 – 172.

Carter, G. A. (1994). Ratios of leaf reflectances in narrow wavebands as indicators of plant stress. *International Journal of Remote Sensing*, 15(3):697–703.

Chappelle, E. W., Kim, M. S., and McMurtrey, J. E. (1992). Ratio analysis of reflectance spectra (rars) - An algorithm for the remote estimation of the concentrations of chlorophyll-a, chlorophyll-b, and carotenoids in soybean leaves. *Remote Sensing of Environment*, 39(3):239–247.

Chen, J. M. (1996). Evaluation of vegetation indices and a modified simple ratio for boreal applications. *Canadian Journal of Remote Sensing*, 22:229–242.

Cho, M. A. and Skidmore, A. K. (2006). A new technique for extracting the red edge position from hyperspectral data: The linear extrapolation method. *Remote Sensing of Environment*, 101(2):181 – 193.

Darvishzadeh, R., Atzberger, C., and Skidmore, A. (2006). Hyperspectral vegetation indices for estimation of leaf area index. In *ISPRS Commission VII Symposium on 'Remote Sensing: From Pixels to Processes.* International Institute for Geo-Information Science and Earth Observation.

Dash, J. and Curran, P. J. (2004). The MERIS terrestrial chlorophyll index. *International Journal of Remote Sensing*, 25(23):5403–5413.

Datt, B. (1999). Visible/near infrared reflectance and chlorophyll content in *Eucalyptus* leaves. *International Journal of Remote Sensing*, 20(14):2741–2759.

Daughtry, C., Walthall, C., Kim, M., de Colstoun, E., and III, J. M. (2000). Estimating corn leaf chlorophyll concentration from leaf and canopy reflectance. *Remote Sensing of Environment*, 74(2):229 – 239.

Elvidge, C. D. and Chen, Z. K. (1995). Comparison of broad-band and narrow-band red and near-infrared vegetation indexes. *Remote Sensing of Environment*, 54(1):38–48.

Filella, I. and Peñuelas, J. (1994). The red edge position and shape as indicators of plant chlorophyll content, biomass and hydric status. *International Journal of Remote Sensing*, 15(7):1459–1470.

Gamon, J., nuelas, J. P., and Field, C. (1992). A narrow-waveband spectral index that tracks diurnal changes in photosynthetic efficiency. *Remote Sensing of Environment*, 41(1):35 – 44.

Gandia, S., Fernández, G., García, J., and Moreno, J. (2004). Retrieval of vegetation biophysical variables from CHRIS/PROBA data in the SPARC campaign. In *ESA SP*, volume 578, pages 40–48.

Garrity, S. R., Eitel, J. U., and Vierling, L. A. (2011). Disentangling the relationships between plant pigments and the photochemical reflectance index reveals a new approach for remote estimation of carotenoid content. *Remote Sensing of Environment*, 115(2):628 – 635.

Gitelson, A., Buschmann, C., and Lichtenthaler, H. (1999). The chlorophyll fluorescence ratio F735/F700 as an accurate measure of the chlorophyll content in plants - Experiments with autumn chestnut and maple leaves. *Remote Sensing of Environment*, 69(3):296–302.

Gitelson, A. and Merzlyak, M. N. (1994). Quantitative estimation of chlorophyll-a using reflectance spectra: Experiments with autumn chestnut and maple leaves. *Journal of Photochemistry and Photobiology B: Biology*, 22(3):247 – 252.

Gitelson, A., Y, G., and MN., M. (2003). Relationships between leaf chlorophyll content and spectral reflectance and algorithms for non-destructive chlorophyll assessment in higher plant leaves. *Journal of Plant Physiology*, 160(3):271–282.

Gitelson, A. A., Kaufman, Y. J., and Merzlyak, M. N. (1996). Use of a green channel in remote sensing of global vegetation from EOS-MODIS. *Remote Sensing of Environment*, 58(3):289 – 298.

Gitelson, A. A. and Merzlyak, M. N. (1997). Remote estimation of chlorophyll content in higher plant leaves. *International Journal of Remote Sensing*, 18(12):2691–2697.

Guyot, G. and Baret, F. (1988). Utilisation de la haute resolution spectrale pour suivre l'etat des couverts vegetaux. In Guyenne, T. D. and Hunt, J. J., editors, *Spectral Signatures of Objects in Remote Sensing*, volume 287 of *ESA Special Publication*, pages 279–286.

Haboudane, D., Miller, J. R., Tremblay, N., Zarco-Tejada, P. J., and Dextraze, L. (2002). Integrated narrow-band vegetation indices for prediction of crop chlorophyll content for application to precision agriculture. *Remote Sensing of Environment*, 81(2-3):PII S0034–4257(02)00018–4.

Hernández-Clemente, R., Navarro-Cerrillo, R. M., Suárez, L., Morales, F., and Zarco-Tejada, P. J. (2011). Assessing structural effects on PRI for stress detection in conifer forests. *Remote Sensing of Environment*, 115(9):2360 – 2375.

Hernández-Clemente, R., Navarro-Cerrillo, R. M., and Zarco-Tejada, P. J. (2012). Carotenoid content estimation in a heterogeneous conifer forest using narrow-band indices and PROSPECT + DART simulations. *Remote Sensing of Environment*, 127(0):298 – 315.

Huete, A. (1988). A soil-adjusted vegetation index (SAVI). *Remote Sensing of Environment*, 25:295–309.

Huete, A., Liu, H., Batchily, K., and van Leeuwen, W. (1997). A comparison of vegetation indices over a global set of TM images for EOS-MODIS. *Remote Sensing of Environment*, 59(3):440 – 451.

Hunt, E. R., Doraiswamy, P. C., McMurtrey, J. E., Daughtry, C. S. T., Perry, E. M., and Akhmedov, B. (2013). A visible band index for remote sensing leaf chlorophyll content at the canopy scale. *International Journal of Applied Earth Observation and Geoinformation*, 21:103–112.

Jordan, C. F. (1969). Derivation of leaf-area index from quality of light on forest floor. *Ecology*, 50(4):663–&.

Kim, M., Daughtry, C., Chappelle, E., McMurtrey, J., and Walthall, C. (1994). The use of high spectral resolution bands for estimating absorbed photosynthetically active radiation (Apar). In *Proceedings of the Sixth Symposium on Physical Measurements and Signatures in Remote Sensing*, pages 299–306, Val D'Isere, France.

Kycko, M., Zagajewski, B., Lavender, S., and Dabija, A. (2019). In situ hyperspectral remote sensing for monitoring of alpine trampled and recultivated species. *Remote Sensing*, 11(11):1296.

le Maire, G., Francois, C., and Dufrene, E. (2004). Towards universal broad leaf chlorophyll indices using PROSPECT simulated database and hyperspectral reflectance measurements. *Remote Sensing of Environment*, 89(1):1–28.

le Maire, G., François, C., Soudani, K., Berveiller, D., Pontailler, J.-Y., Bréda, N., Genet, H., Davi, H., and Dufrêne, E. (2008). Calibration and validation of hyperspectral indices for the estimation of broadleaved forest leaf chlorophyll content, leaf mass per area, leaf area index and leaf canopy biomass. *Remote Sensing of Environment*, 112(10):3846 – 3864.

Lichtenthaler, H., Lang, M., Sowinska, M., Heisel, F., and Miehé, J. (1996). Detection of vegetation stress via a new high resolution fluorescence imaging system. *Journal of Plant Physiology*, 148(5):599–612.

Maccioni, A., Agati, G., and Mazzinghi, P. (2001). New vegetation indices for remote measurement of chlorophylls based on leaf directional reflectance spectra. *Journal of Photochemistry and Photobiology B: Biology*, 61(1-2):52 – 61.

McMurtrey, J. E., Chappelle, E. W., Kim, M. S., Meisinger, J. J., and Corp, L. A. (1994). Distinguishing nitrogen-fertilization levels in-field corn (*Zea mays* L) with actively induced fluorescence and passive reflectance measurements. *Remote Sensing of Environment*, 47(1):36–44.

Meiforth, J. J., Buddenbaum, H., Hill, J., and Shepherd, J. (2020). Monitoring of canopy stress symptoms in new zealand kauri trees analysed with AISA hyperspectral data. *Remote Sensing*, 12(6):926.

Merzlyak, M. N., Gitelson, A. A., Chivkunova, O. B., and Rakitin, V. Y. (1999). Non-destructive optical detection of pigment changes during leaf senescence and fruit ripening. *Physiologia Plantarum*, 106(1):135–141.

MILLER, J. R., HARE, E. W., and WU, J. (1990). Quantitative characterization of the vegetation red edge reflectance 1. an inverted-gaussian reflectance model. *International Journal of Remote Sensing*, 11(10):1755–1773.

Montesinos-López, O. A., Montesinos-López, A., Crossa, J., de los Campos, G., Alvarado, G., Suchismita, M., Rutkoski, J., González-Pérez, L., and Burgueño, J. (2017). Predicting grain yield using canopy hyperspectral reflectance in wheat breeding data. *Plant Methods*, 13(1).

Oppelt, N. and Mauser, W. (2004). Hyperspectral monitoring of physiological parameters of wheat during a vegetation period using AVIS data. *International Journal of Remote Sensing*, 25(1):145–159.

Peñuelas, J., Baret, F., and Filella, I. (1995). Semiempirical indexes to assess carotenoids chlorophyll-a ratio from leaf spectral reflectance. *Photosynthetica*, 31(2):221–230.

Peñuelas, J., Gamon, J. A., Fredeen, A. L., Merino, J., and Field, C. B. (1994). Reflectance indexes associated with physiological-changes in nitrogen-limited and water-limited sunflower leaves. *Remote Sensing of Environment*, 48(2):135–146.

Pearson, R. L. and Miller, L. D. (1972). Remote Mapping of Standing Crop Biomass for Estimation of the Productivity of the Shortgrass Prairie. In *Remote Sensing of Environment, VIII*, page 1355.

Peng, Y., Fan, M., Wang, Q., Lan, W., and Long, Y. (2018). Best hyperspectral indices for assessing leaf chlorophyll content in a degraded temperate vegetation. *Ecology and Evolution*, 8(14):7068–7078.

PEN¯UELAS, J., FILELLA, I., LLORET, P., MUN¯OZ, F., and VILAJELIU, M. (1995). Reflectance assessment of mite effects on apple trees. *International Journal of Remote Sensing*, 16(14):2727–2733.

Qi, J., Chehbouni, A., Huete, A., Kerr, Y., and Sorooshian, S. (1994). A modified soil adjusted vegetation index. *Remote Sensing of Environment*, 48(2):119 – 126.

Rondeaux, G., Steven, M., and Baret, F. (1996). Optimization of soil-adjusted vegetation indices. *Remote Sensing of Environment*, 55(2):95–107.

Roujean, J. L. and Breon, F. M. (1995). Estimating par absorbed by vegetation from bidirectional reflectance measurements. *Remote Sensing of Environment*, 51(3):375–384.

Sims, D. and Gamon, J. (2002). Relationships between leaf pigment content and spectral reflectance across a wide range of species, leaf structures and developmental stages. *Remote Sensing of Environment*, 81(2):337–354.

Smith, R., Adams, J., Stephens, D., and Hick, P. (1995). Forecasting wheat yield in a mediterranean-type environment from the NOAA satellite. *Australian Journal of Agricultural Research*, 46(1):113–125.

Tucker, C. J. (1979). Red and photographic infrared linear combinations for monitoring vegetation. *Remote Sensing of Environment*, 8(2):127–150.

Velichkova, K. and Krezhova, D. (2019). COMPARATIVE ANALYSIS OF HYPERSPECTRAL VEGETATION INDICES FOR REMOTE ESTIMATION OF LEAF CHLOROPHYLL CONTENT AND PLANT STATUS. *RAD Association Journal*, 3(3).

Vincini, M., Frazzi, E., and D'Alessio, P. (2006). Angular dependence of maize and sugar beet VIs from directional CHRIS/PROBA data. In *Fourth ESA CHRIS PROBA Workshop. ESRIN*, pages 19–21, Frascati, Italy.

Vogelmann, J. E., Rock, B. N., and Moss, D. M. (1993). Red edge spectral measurements from sugar maple leaves. *International Journal of Remote Sensing*, 14(8):1563–1575.

Wang, L. and Wei, Y. (2016). Revised normalized difference nitrogen index (NDNI) for estimating canopy nitrogen concentration in wetlands. *Optik*, 127(19):7676–7688.

Wu, C., Niu, Z., Tang, Q., and Huang, W. (2008). Estimating chlorophyll content from hyperspectral vegetation indices: Modeling and validation. *Agricultural and Forest Meteorology*, 148(8-9):1230 – 1241.

Wu, W. (2014). The generalized difference vegetation index (GDVI) for dryland characterization. *Remote Sensing*, 6(2):1211–1233.

Zarco-Tejada, P. J., Gonzalez-Dugo, V., Williams, L. E., Suarez, L., Berni, J. A. J., Goldhamer, D., and Fereres, E. (2013). A PRI-based water stress index combining structural and chlorophyll effects: Assessment using diurnal narrow-band airborne imagery and the CWSI thermal index. *Remote Sensing of Environment*, 138:38–50.

Zarco-Tejada, P. J. and Miller, J. R. (1999). Land cover mapping at BOREAS using red edge spectral parameters from CASI imagery. *Journal of Geophysical Research-atmospheres*, 104(D22):27921–27933.

ZARCOTEJADA, P., BERJON, A., LOPEZLOZANO, R., MILLER, J., MARTIN, P., CACHORRO, V., GONZALEZ, M., and DEFRUTOS, A. (2005). Assessing vineyard condition with hyperspectral indices: Leaf and canopy reflectance simulation in a row-structured discontinuous canopy. *Remote Sensing of Environment*, 99(3):271–287.

Zhen, Z., Yunsheng, L., Moses, O. A., Rui, L., Li, M., and Jun, L. (2020). Hyperspectral vegetation indexes to monitor wheat plant height under different sowing conditions. *Spectroscopy Letters*, 53(3):194–206.
